# Pemetrexed single agent chemotherapy in previously treated patients with locally advanced or metastatic non-small cell lung cancer

**DOI:** 10.1186/1471-2407-8-216

**Published:** 2008-07-31

**Authors:** Francesca Russo, Alessandra Bearz, Gianni Pampaloni, the investigators of the Italian Pemetrexed monotherapy of NSCLC group

**Affiliations:** 1 Medical Department, Eli Lilly Italia S.p.A. Sesto Fiorentino (FI), Italy; 2 Oncologia Medica, Centro di Riferimento Oncologico Aviano (PN), Italy

## Abstract

**Background:**

The main objective of this study was to evaluate the safety of second-line pemetrexed in Stage IIIB or IV NSCLC.

**Methods:**

Overall, 95 patients received pemetrexed 500 mg/m^2 ^i.v. over Day 1 of a 21-day cycle. Patients also received oral dexamethasone, oral folic acid and i.m. vitamin B12 supplementation to reduce toxicity. NCI CTC 2.0 was used to rate toxicity. All the adverse events were graded in terms of severity and relation to study treatment. Dose was reduced in case of toxicity and treatment was delayed for up to 42 days from Day 1 of any cycle to allow recovering from study drug-related toxicities. Tumor response was measured using the RECIST criteria.

**Results:**

Patients received a median number of 4 cycles and 97.8% of the planned dose. Overall, 75 patients (78.9% of treated) reported at least one adverse event: 34 (35.8%) had grade 3 as worst grade and only 5 (5.2%) had grade 4. Drug-related events occurred in 57.9% of patients. Neutropenia (8.4%) and leukopenia (6.3 %) were the most common grade 3/4 hematological toxicities. Grade 3 anemia and thrombocytopenia were reported in 3.2% and 2.1% of patients, respectively. Diarrhea (6.3%), fatigue (3.2%) and dyspnea (3.2%) were the most common grade 3/4 non-hematological toxicities. The most common drug-related toxicities (any grade) were pyrexia (11.6%), vomiting, nausea, diarrhea and asthenia (9.5%) and fatigue (8.4%). Tumor Response Rate (CR/PR) in treated patients was 9.2%. The survival at 4.5 months (median follow-up) was 79% and the median PFS was 3.1 months. Twenty patients (21.1%) died mainly because of disease progression.

**Conclusion:**

Patients with locally advanced or metastatic NSCLC could benefit from second-line pemetrexed, with a low incidence of hematological and non-hematological toxicities.

## Background

Lung cancer is more often diagnosed and is by far the most common cause of death from cancer in both genders worldwide [1,2]. Almost 80% of lung cancers can be classified as Non Small Cell Lung Cancer (NSCLC), with 65% to 75% of cases presenting as locally advanced (Stage III) or metastatic disease (Stage IV) [3,4].

Significant improvements in median survival in advanced NSCLC patients have been achieved with the use of platinum-based chemotherapy [5], particularly in patients with good performance status, and with newer cytotoxic agents, such as gemcitabine, paclitaxel, docetaxel or vinorelbine [6,7]. It is actually believed that the next significant advance in the treatment of NSCLC might derive from the use of targeted agents, as monotherapy or in combination with standard chemotherapy regimens, without increasing toxicity [6].

Pemetrexed is a new multi-target antifolate agent approved for the treatment of malignant pleural mesothelioma and NSCLC. Pemetrexed exerts its cytotoxic effect through inhibition of Thymidylate Synthase, Dihydrofolate Reductase and Glycinamide Ribonucleotide Formyl Transferase [8], which are involved in DNA synthesis and folate metabolism [9]. The multiple inhibitions of several key folate-requiring enzymes may account both for the antitumor activity and the potential cytotoxic effect of pemetrexed. It has been found that the hematological and non-hematological toxicities of pemetrexed can be reduced through routine vitamin supplementation (folic acid and vitamin B12), without loss of efficacy [10].

Several reports have documented the effects of pemetrexed given as a single agent and in combination in first- or second-line chemotherapy in advanced NSCLC [11]. In phase II trials, pemetrexed has shown high efficacy and favorable toxicity when given in combination with platinum agents, gemcitabine and vinorelbine [12,13]. A recent phase III trial that compared pemetrexed with docetaxel in previously treated NSCLC patients showed equivalent efficacy in response rate and survival, and significantly less toxicity in the pemetrexed group when compared to docetaxel [14].

According to Italian legislation, which establishes a drug dispensing as 'therapeutic use' prior to approval for use in local market, this study was aimed at extending the clinical experience with pemetrexed in pretreated patients with locally advanced or metastatic NSCLC.

## Methods

### Patients

Adult patients of both genders with locally advanced or metastatic NSCLC (Stage IIIB or IV at entry), previously treated with no more than two chemotherapy regimens for advanced disease, were eligible for the study. Prior chemotherapy and/or radiotherapy (excluding pemetrexed) were to be completed at least 2 weeks prior to study enrollment and the patients should have recovered from any acute toxic effect of previous therapy. Prior radiation therapy allowed to < 25% of the bone marrow. Moreover, eligible patients were required to have a ECOG Performance Status 0 to 2, an estimated life expectation of at least 8 weeks, and an adequate bone marrow reserve. Patients with evidence of hepatic or renal insufficiency, active infection, inability to take folic acid, vitamin B12 supplementation or corticosteroids, signs of malnourishment or > 10% weight loss in the past 6 weeks, or others serious concomitant disorders (including oncologic emergencies) were excluded from the study. Pregnant or breastfeeding females were also not allowed to taking part in the study, as well as an adequate contraceptive method was to be used for the whole study duration. Patients were to be discontinued from the study in the case of evidence of progressive disease or unacceptable toxicity despite dose adjustment.

The participant patients gave their written informed consent prior to enter in the study. The study protocol and the informed consent form were reviewed and approved by the Independent Ethics Committees of each participating center prior to any study-related procedure was started.

### Treatments

Pemetrexed 500 mg/m^2 ^(Alimta^®^, Eli Lilly and Company, Indianapolis, IN) was administered i.v. over approximately 10 minutes on Day 1 of a 21-day cycle. Dexamethasone 4 mg or equivalent corticosteroid was taken orally twice daily on the day before, the day of, and the day after each dose of pemetrexed. Folic acid supplementation 350 to 600 μg or equivalent was taken orally daily beginning approximately 1 to 2 weeks prior to the first dose of pemetrexed and continued until 3 weeks after study therapy discontinuation. Patients also received a 1000 μg vitamin B12 i.m. injection approximately 1 to 2 weeks prior to the first dose of pemetrexed, to be repeated approximately every 9 weeks until 3 weeks after study therapy discontinuation.

Any patient who required a pemetrexed dose reduction due to hematological or non-hematological toxicities was treated further according to dose reductions. Any patient requiring > 2 reductions due to toxicity was to be withdrawn from study therapy. Treatment could have been delayed for up to 42 days from Day 1 of any cycle to allow recovering from study drug-related toxicities.

No other chemotherapy, immunotherapy, hormonal cancer therapy, radiation therapy, surgery for cancer, or any other experimental medications was permitted during the study. Disease progression requiring alternative antitumor treatment led to early discontinuation of study therapy. If patient required radiotherapy treatment (both palliative or not) during the study, pemetrexed was discontinued until 2 weeks after the completion of radiation treatment.

The use of growth factors was not allowed by study protocol.

### Outcome measures

The analysis of safety was the primary endpoint of the study. The safety measures used in the study included adverse events, physical examinations and clinical laboratory tests (hematology, blood chemistry and urinary creatinine clearance). All the adverse events were evaluated in terms of severity and relation to study treatment, while toxicities according to the National Cancer Institute Common Toxicity Criteria (NCI CTC) version 2.0 [15].

The evaluation of the best tumor response rate was performed at the end of the treatment period and the Response Evaluation Criteria in Solid Tumors (RECIST) were recommended [16]. The progression free survival (PFS) was the time from study entry to disease progression or death, while the overall survival time was defined as the time from study entry to death due to any cause. Investigators followed-up the survival status of patients who had discontinued study therapy.

Adverse events were considered those emerging during treatment or present at baseline and worsening during the study.

### Statistics

The statistical analysis was performed using the SAS version 8.2 (Cary, NC, US). The analyses were mainly descriptive: summary statistics were given for patient characteristics, treatment administration and all safety variables (laboratory tests and adverse events). Adverse events were coded using the MedDRA dictionary. Tumor Response Rate (complete response/partial response [CR/PR]) was calculated considering all patients who received at least one dose of study drug. PFS and overall survival time were analyzed by means of Kaplan-Meier method.

## Results

A total of 102 patients were enrolled in 35 Italian centers from December 2004 to May 2005. The demographic and baseline clinical condition of treated patients are summarized in Table 1. Most of the patients were in good Performance Status: 87 patients (93.6% of valuable patients) were ECOG PS 0 to 1. Adenocarcinoma/Neoplasia NOS was the most frequent histological type, representing approximately half of cases.

**Table 1:** Demographic and baseline clinical condition of the treated patients (n = 95)

Age, *years*: mean ± SD (range)	62.4 ± 10.6 (25–82)
Age ranges, N (%):	
≤ 50	11 (11.6)
51–60	21 (22.1)
61–70	44 (46.3)
> 70	19 (20.0)
Sex: N (%)	
Males	72 (75.8)
Females	23 (24.2)

Weight, *kg*: mean ± SD (range)	72.2 ± 13.6 (41–110)

NSCLC, *histological type*: N (%)	
Neoplasia NOS, adenocarcinoma	46 (48.4)
Squamous cells	26 (27.4)
Large cells	2 (2.1)
Other	21 (22.1)

ECOG, *score*: N (%)	
0	58 (62.4)
1	29 (31.2)
2	5 (5.4)
3	1 (1.1)
Not available	2

N = number of patients, % refers to total of treated patients with available data

Ninety-five of them (93.1% of enrolled) received at least one dose of study drug. Seven patients were included but did not receive study drug (2 because of physician decision, 2 patient decision, 2 deaths, 1 entry criteria violation).

The median received cycles was 4.0 (range 1–15), while the median number of weeks of treatment was 12.1 (range 1.4–57.3). Fifty patients (52.6%) had dose modification at least in one cycle: pemetrexed dose was reduced due to adverse events in 12 patients and was delayed (mostly due to adverse events or conflict in scheduling) in 48 patients. The median relative dose intensity was 97.8% (range 63.1–104.0). Deviations from the scheduled dosing of dexamethasone, folic acid and vitamin B12 were reported in 3, 7 and 8 patients, respectively.

The main reasons for treatment discontinuation were lack of efficacy (46 patients, 48.4%), physician decision (13, 13.7%), objective responses (13, 13.7%) and patient decision (8, 8.4%). Fifteen patients had protocol violation and the most common was the incorrect dose reduction due to toxicity (7 patients).

### Safety

Seventy-five patients (78.9% of treated) reported at least one adverse event during the study, 34 patients (35.8%) and 5 patients (5.2%) experienced grade 3 and grade 4 adverse events, respectively. Fifty-five patients (57.9%) had adverse events considered by physicians as possibly related to study treatment.

Table 2 shows adverse events reported in ≥ 5% of patients by preferred term and study drug relationship. The most common adverse events were pyrexia (reported in 26.3% of treated patients and judged as drug-related in 11.6%), asthenia (overall 13.7% of patients, drug-related in 9.5%) and dyspnea (overall 11.6% of patients, drug-related in only one case). General disorders and administration site conditions (26.3%), gastrointestinal disorders (23.2%) and blood and lymphatic system disorders (22.1%) were the system organ classes with the highest incidence of adverse events related to pemetrexed.

**Table 2:** Treatment-emergent adverse events reported by ≥ 5% of treated patients by preferred term and study drug relationship: data are number of patients with rates in brackets (N = 95)

	All Causalities	Treatment Related
Patients with ≥ 1 adverse event	75 (78.9)	55 (57.9)

Pyrexia	25 (26.3)	11 (11.6)
Asthenia	13 (13.7)	9 (9.5)
Dyspnea	11 (11.6)	1 (1.1)
Neutropenia	10 (10.5)	10 (10.5)
Vomiting	10 (10.5)	9 (9.5)
Diarrhea	10 (10.5)	9 (9.5)
Anemia	10 (10.5)	8 (8.4)
Nausea	9 (9.5)	9 (9.5)
Fatigue	9 (9.5)	8 (8.4)
Cough	8 (8.4)	3 (3.2)
Anorexia	6 (6.3)	1 (1.1)
Leucopenia	5 (5.3)	5 (5.3)
Mucosal inflammation	5 (5.3)	5 (5.3)
Thrombocytopenia	5 (5.3)	5 (5.3)
Rash	5 (5.3)	4 (4.2)
Chest pain	5 (5.3)	1 (1.1)
Peripheral Edema	5 (5.3)	1 (1.1)

The highest incidences of CTC grade 3/4 adverse events were reported as blood and lymphatic system disorders (17.9%), gastrointestinal disorders (9.5%) and general disorders and administration site conditions (9.5%). Grade 3 adverse events reported in > 1 patient included anemia (3 patients), leukopenia (6), neutropenia (6), thrombocytopenia (2), diarrhea (6), nausea (2), vomiting (2), fatigue (3), mucosal inflammation (2), thrombocytopenia (2), and dyspnea (3 patients). Grade 4 adverse events included neutropenia (2 patients), and acute myocardial infarction, myocardial ischemia and melaena (all occurred in the same patient).

A total of 20 patients (21.1% of treated population) had at least one event fulfilling the criteria for a serious adverse event; 5 of them were considered drug-related (neutropenia in 2 patients, diarrhea, pyrexia, melaena, anemia and vomiting in 1). Overall, 19 patients (20.0%) died due to disease progression: 5 patients (5.3%) died while on treatment or within 30 days of treatment discontinuation, 14 died after 30 days from treatment discontinuation. One patient died due to cardiac failure.

Hematological assessments were performed on 90 out the 95 treated patients. Table 3 shows the out of range hematological values observed during treatment (NCIC-CTC grading). NCIC-CTC grade 3 hematological toxicities were the following: anemia 2.2% of patients, leucopenia 17.8%, neutropenia 18.9%, and thrombocytopenia 4.4%.

**Table 3:** Hematology abnormalities observed during treatment (worst NCIC-CTC grading): data are number of patients with rates in brackets (N = 90)

Laboratory Parameter	Any grade ≥ 1	Grades 3–4
Hemoglobin	63 (70.0)	2 (2.2)
Neutrophils	50 (55.6)	17 (18.9)
Platelets	37 (41.1)	4 (4.4)
WBCs	57 (63.3)	16 (17.8)

No clinically relevant changes in vital signs were reported during the study.

### Efficacy

Table 4 shows the results of the overall tumor best response in the treated patients population with measurable disease at baseline (N = 87): 8 patients (9.2%; 95%: 4.1 to 17.3) were responders (1 CR and 7 PR), 23 patients (26.4%) were stable on their disease, 49 patients (56.3%) had disease progression as best response and 7 patients (8.0%) were not evaluable for response.

**Table 4:** Results of the overall tumor response in the treated population: data are number of patients with rates in brackets

Best Overall Tumor Response	Treated Population (N = 87)*
Complete Response (CR)	1 (1.1)
Partial Response PR)	7 (8.0)
Response Rate (CR + PR)	8 (9.2)
Stable Disease/No Response (SD)	23 (26.4)
Progressive Disease (PD)	49 (56.3)
Not Evaluable	7 (8.0)

*Numbers and rates refer to the amount of patients assessed for tumor response (response was not available in 8 patients in the treated population)

The Kaplan-Meier survival analysis at 4.5 months (median follow-up) was 79% (95% CI: 71 to 88%). The median PFS was 3.1 months (95% C.I. 2.4 to 3.8).

## Discussion

Previous phase 2 studies have indicated that pemetrexed (Alimta^®^) has clinical activity in NSCLC. A comparative trial of Pemetrexed and docetaxel (Eli Lilly Protocol H3E-MC-JMEI), compared 571 patients with locally advanced or metastatic NSCLC who had previously been treated with chemotherapy.

The primary objective of this study was to confirm the safety profile of pemetrexed (500 mg/m^2 ^dose, day 1 of a 21-day cycle) as second line treatment in patients with locally advanced or metastatic (Stage IIIB or IV) NSCLC. Pemetrexed was supplemented with dexamethasone, folic acid and vitamin B12 was given every 21 days. This regimen is recommended based on previous experiences [17,14], which showed a significant improved tolerance when pemetrexed is given with corticosteroids and vitamins supplementation.

The secondary objective of the study was to assess the response rate in patients with measurable disease according to the RECIST criteria.

In this study 95 patients were examined. The majority of patients (>90%) had good clinical conditions (ECOG PS 0 or 1). The median number of cycles received was 4 and the median number of weeks of treatment was 12.1. Pemetrexed was well tolerated. The safety profile of pemetrexed did not differ from what observed in previous phase I/II studies and in the large phase III study comparing pemetrexed and docetaxel as second-line treatment in locally advanced or metastatic NSCLC [14]. In the latter trial, which led to the regulatory approval of pemetrexed as monotherapy for the second-line treatment of NSCLC, the incidence of hematological toxicities (e.g. grade 3/4 neutropenia, febrile neutropenia, and neutropenia with infections) and other drug-related adverse events was significantly lower with pemetrexed than with docetaxel. The results of the present study confirm the favorable toxicity profile of pemetrexed when given over 500 mg/m^2 ^and supplemented by vitamin B12 and folic acid.

Vitamin supplementation significantly reduces the incidence of grade 3–4 hematological toxicity, as shown in a previous trial comparing pemetrexed administered with or without vitamins [18].

The most frequent hematological toxicities were neutropenia and anemia (any grade) and the most frequent non-hematological toxicities were pyrexia, fatigue and dyspnea (any grade).

In the population of patients with measurable disease at baseline the observed response rate was 9.2% and it was similar to the Response Rate reported in the randomized phase III study (8.8%) when pemetrexed was compared to docetaxel [14].

It is generally agreed that response rate cannot be taken as indicator of clinical benefit in pretreated patients with locally advanced or metastatic NSCLC and the relationship between response rate and improved survival is unclear, so that response rate cannot be considered as a surrogate endpoint. However, a prolonged survival in pretreated advanced NSCLC patients has been observed, in spite of a response rate lower than 10%. This therefore suggests a possible contribution from cytotoxic agents to disease stabilization and to the clinical benefit observed [19].

In our study, the survival at approximately 4 months (median follow-up time) was 79% and the median progression-free survival was 3.1 months, what is in line with the reported survival rate in the reported phase III trial by Hanna et al. in the comparative study vs Docetaxel [14].

It is well known that, especially in 2^nd ^line, tolerability and toxicity profile of a cytotoxic combination might influence the choice of treatment, even when the efficacy parameters of possible therapies (e.g. survival, progression free survival and response rate) are similar. The duration of infusion, schedule administration and patients acceptance should be also taken into consideration for the choice of a regimen. When compared to the other agents currently approved for 2^nd ^line treatment in NSCLC, the 10-minutes infusion time of pemetrexed over Day 1 of a 21-day cycle might increase the convenience of the treatment and patient compliance.

Therefore, the study confirmed that pretreated patients with locally advanced or metastatic NSCLC will likely benefit from single-agent pemetrexed treatment (with vitamin supplementation), with an additional advantage in decreasing hematological (including febrile neutropenia) and non-hematological toxicities.

Since, due to a minor flaw in the original study design, there are no available data on whether patients were treated with pemetrexed in 2^nd ^or 3^rd ^line, it is not possible to assess any correlation between the number of previous lines of treatment and response to pemetrexed.

It has been recently pointed out that the administration of Pemetrexed in combination with other agents (eg. Cisplatin, Carboplatin or gemcitabine) in the treatment of advanced NSCLC may provide further clinical benefits caused by its particular mode of action when blocking intracellular three enzymes system. A deeper knowledge about those enzyme system (eg: TS) may be used in future to identify patients responders to pemetrexed [20]. The use of targeted compounds to specific molecular pathways, given in addition to standard chemotherapy regimens, might represent the next step in the treatment of NSCLC and overall characteristics of pemetrexed makes it a candidate in a tailored therapies context.

The present study contributes to provide even more information on clinical experience with pemetrexed and further prospective randomized clinical trials will confirm pemetrexed (single agent or in combination) as a valid option for pretreated locally advanced or metastatic NSCLC patients.

## Competing interests

The study was fully sponsored by Eli Lilly Italia.  Francesca Russo and Gianni Pampaloni are employed at Eli Lilly Italia.

## Authors' contributions

All authors have given substantial contributions to conception and design the study. FR and GP have given substantial contributions to analysis and interpretation of data, and in the revision of the manuscript. AB has given relevant contributions in the recruitment of patients, in the critical revision of the manuscript, and in final approval prior to publication. All authors read and approved the final manuscript.

## Pre-publication history

The pre-publication history for this paper can be accessed here:


